# Reliability, validity and responsiveness of E-RS:COPD in patients with spirometric asthma-COPD overlap

**DOI:** 10.1186/s12931-019-1070-6

**Published:** 2019-05-31

**Authors:** Linda M. Nelsen, Laurie A. Lee, Wei Wu, Xiwu Lin, Lindsey Murray, Steven J. Pascoe, Nancy K. Leidy

**Affiliations:** 1 Value Evidence and Outcomes, GSK, Collegeville, PA 19426 USA; 2 Research and Development, GSK, Stevenage, Hertfordshire, UK; 3 Biostatistics, PAREXEL International, Research Triangle Park, Raleigh, NC USA; 4 Patient-Centered Research, Evidera, Bethesda, MD USA; 5 Respiratory Medicines Development Center, GSK, Research Triangle Park, Raleigh, NC USA

**Keywords:** Asthma-COPD overlap, Post hoc, Respiratory symptoms, E-RS:COPD, Wheeze

## Abstract

**Background:**

The Evaluating Respiratory Symptoms in Chronic Obstructive Pulmonary Disease (E-RS:COPD) is a patient-reported diary that assesses respiratory symptoms in stable COPD.

**Methods:**

This post hoc analysis of a randomized, double-blind, parallel-arm trial (GSK ID: 200699; NCT02164539) assessed the structure, reliability, validity and responsiveness of the E-RS, and a separate wheeze item, for use in patients with a primary diagnosis of asthma or COPD, but with spirometric characteristics of both (fixed airflow obstruction and reversibility to salbutamol; a subset of patients referred to as spirometric asthma-COPD overlap [ACO]; *N* = 338).

**Results:**

Factor analysis demonstrated that E-RS included Cough and Sputum, Chest Symptoms, and Breathlessness domains, with a Total score suitable for quantifying overall respiratory symptoms (comparative fit index: 0.9), consistent with the structure shown in COPD. The wheeze item did not fit the model. Total and domain scores were internally consistent (Cronbach’s alpha: 0.7–0.9) and reproducible (intra-class correlations > 0.7). Moderate correlations between RS-Total and RS-Breathlessness scores were observed with St George’s Respiratory Questionnaire (SGRQ) Total and Activity domain scores at baseline (r = 0.43 and r = 0.48, respectively). E-RS scores were sensitive to change when a patient global impression of change and SGRQ change scores were used to define responders, with changes of ≥ − 1.4 in RS-Total score interpreted as clinically meaningful.

**Conclusions:**

E-RS:COPD scores were reliable, valid and responsive in this sample, suggesting the measure may be suitable for evaluating the severity of respiratory symptoms and the effects of treatment in patients with asthma and COPD that exhibit spirometric characteristics of both fixed airflow obstruction and reversibility. Further study of this instrument and wheeze in new samples of patients with ACO is warranted.

**Electronic supplementary material:**

The online version of this article (10.1186/s12931-019-1070-6) contains supplementary material, which is available to authorized users.

## Background

Patients with features of both asthma and chronic obstructive pulmonary disease (COPD) may present with chronic airflow obstruction and a reversible component. Asthma-COPD overlap (ACO) [1, 2] was first considered in 2009 [3] before appearing in the Global Initiative for Chronic Obstructive Lung Disease (GOLD) strategic document in 2014. [4] Its prevalence can vary depending on the definition used. [5–7] Clinical studies have used varying diagnostic criteria for ACO, [8] and while recent efforts to identify recognized criteria for the condition have been made by GOLD/Global Initiative for Asthma (GINA) in 2015 [9], GINA in 2018 [1], and an international panel of experts in 2016, [10] there is no definitive consensus definition for ACO.

Although pharmacologic treatments have been approved in both asthma and COPD, little is known about their effects in patients with ACO. In addition, there are currently no ACO-specific patient-reported outcome (PRO) measures available to measure treatment effects on respiratory symptoms in ACO. Patients with ACO report similar symptoms to patients with COPD or asthma, [5, 11–13] including shortness of breath, cough, wheezing, difficulty breathing, mucus/phlegm and chest tightness. [5, 11–13] A reliable and valid PRO measure will provide important information on the respiratory symptom burden of ACO and the effects of treatment in this patient population. Based on the similarities in symptoms between COPD and ACO and their shared underlying airflow limitation, symptom measures developed for COPD may be appropriate in ACO. Among the most frequently utilized PROs in COPD is the St George’s Respiratory Questionnaire (SGRQ), which is a 50-item, self-administered measure for evaluating health status, with a subscale measuring the severity and impact of symptoms. [14] This instrument was not developed to evaluate respiratory symptoms per se, but includes an assessment of this as part of an overall health status score. Further, the SGRQ was designed for periodic rather than daily administration, with the latter considered optimal to capture patient symptoms more precisely. Other health status measures, such as the COPD Assessment Test [15] and the Chronic Respiratory Questionnaire [16] include symptom questions but are not scored to evaluate each of the cardinal symptoms of COPD. Questionnaires such as the University of California San Diego Shortness of Breath Questionnaire [17] and the Cough and Sputum Assessment Questionnaire [18] evaluate specific symptoms, i.e. dyspnea and cough and sputum, respectively. However, none of these instruments measure all of the cardinal symptoms, and with a daily recall to support insight into daily symptom fluctuations. The Evaluating Respiratory Symptoms in COPD (E-RS:COPD) (Evidera, Bethesda, MD, USA) measure is a patient-reported diary used to assess the cardinal symptoms of COPD overall and through three symptom-specific domains: Cough and Sputum, Chest Symptoms and Breathlessness. [19, 20] The E-RS includes 11 items from the Exacerbations of Chronic Pulmonary Disease Tool (EXACT) (Evidera, Bethesda, MD, USA) [21] and has shown evidence of content validity, reliability, validity and responsiveness in stable COPD. [19–21] It has also been qualified by the Food and Drug Administration and European Medicines Agency for use as an exploratory endpoint in drug development trials of COPD. [22, 23]

This post hoc analysis of clinical trial data (GSK: 200699, NCT02164539) [24] aimed to assess the factor structure, reliability, validity and responsiveness of the E-RS in patients with a primary diagnosis of asthma or COPD, and spirometric characteristics of both conditions (fixed airflow obstruction and reversibility to salbutamol). The term “spirometric ACO” is used, and in this study it reflects an ACO population inclusive of patients with and without exposure to smoking or biomass fuels. [24] As wheeze is not part of the E-RS, [20] a previously developed wheeze item (GSK, Research Triangle Park, NC, USA) was used to explore the relationship between the E-RS and wheeze scores and determine if a modification to the E-RS:COPD would be needed to use the measure in spirometric ACO.

## Methods

### Trial design

This was an exploratory post hoc analysis of a double- blind, parallel-arm trial conducted in Argentina, Germany, Poland, Romania, Russia, Ukraine and the USA during 2014–2015 (GSK: 200699, NCT02164539). [24] Following a 4-week run-in period (Day − 28 to Day − 1), patients with spirometric ACO were randomized on Day 1 to receive fluticasone furoate 100 mcg alone or in combination with either umeclidinium (15.6, 62.5, 125 or 250 mcg) or vilanterol 25 mcg, once daily for 4 weeks (Day 1 to Day 28) using an Ellipta inhaler (ELLIPTA is owned by or licensed to the GSK group of companies).

The trial was approved by an ethics committee or institutional review board in each country and conducted in accordance with the International Conference on Harmonisation of Technical Requirements for Registration of Pharmaceuticals for Human Use Good Clinical Practice guidelines [25] and the Declaration of Helsinki, 2013. [26] All patients provided written, informed consent.

### Patient sample

This trial enrolled patients with a primary diagnosis of asthma or COPD, and who had spirometric characteristics of both conditions (fixed airflow obstruction and reversibility to salbutamol). [24] Patients were ≥ 18 years of age and had sufficient medical history (either smokers or non-smokers) to receive a diagnosis (via signs and symptoms) of COPD (American Thoracic Society/European Respiratory Society definition) [27] and an asthmatic component evidenced by spirometric criteria described in Additional File 1, which were consistent with the spirometric criteria for ACO available at time of study. [9] Therefore, the patients enrolled in this study were considered as having spirometric ACO. Patients were assigned a primary diagnosis (clinical) of either asthma or COPD, based on medical history and clinical judgement, at the beginning of the run-in period. This reflected the primary component of the patient’s disease. Patients also received a historical diagnosis by a healthcare professional (HCP) based on their medical records and determined by the investigator by asking the questions ‘do you have asthma?’ or ‘do you have COPD?’ at Visit 1.

### Measures

Patients completed the 14-item EXACT [21] and wheeze item using electronic daily diaries throughout the run-in and treatment periods (Day − 28 to Day 28). For this analysis, only the 11 items comprising the E-RS instrument were included. For the wheeze item, patients were also asked “Did you wheeze today?” with response options of ‘Not at all’, ‘Rarely’, ‘Occasionally’, ‘Frequently’ and ‘Almost constantly’.

Morning and evening peak expiratory flow (PEF), morning and evening patient-initiated spirometry and rescue medication use were recorded using electronic daily diaries. Other variables from clinic visits included forced expiratory volume in 1 s (FEV_1_) % predicted (Day − 28, Day 1 and Day 28); the PRO measures of the SGRQ (Day 1 and Day 28); Patient Global Impression of Change (PGIC) in lung condition (overall disease) using a 7-point Likert scale ranging from much better to much worse (Day 28); and modified Medical Research Council (mMRC) dyspnea status (Day − 28 and Day 1).

### Statistical analyses

Analyses were performed on the intent-to-treat population, including all patients who received ≥1 dose of the randomized treatment. [24] Data from the run-in period and the first 4 weeks of treatment were used, pooled across treatment groups. The baseline week was the last week of the run-in period (Day − 7 to Day − 1) and the final treatment week was the last week of the treatment period (Day 22 to Day 28). Exploratory analyses stratified by primary diagnosis were also conducted to assess the extent to which the E-RS and wheeze scores performed differently in asthma- predominant versus COPD-predominant disease.

#### Item analysis

Descriptive statistics for the E-RS items, as well as the Total and domain scores (using the E-RS item- and domain-level scoring algorithm [20]) and the wheeze item, were calculated for Day − 1 (prior to randomization) and Day 28 (end of study treatment). Inter-item correlations were analyzed at Day − 1 using Spearman correlation coefficients. Correlations > 0.40 and > 0.70 were defined as moderate and strong, respectively. [28]

#### Factor analysis

Using data from Day − 1, a confirmatory factor analysis (CFA) using structural equation modeling (higher order three-factor model) was conducted to test the factor structure of the E-RS in relation to that shown in COPD [19, 20], and to identify which factor (domain) each item was most strongly associated with (factor loading). Factor loadings > 0.40 were considered acceptable. An exploratory factor analysis was performed with the wheeze item to assess whether it was associated with one or more of the E-RS domains, suggesting a new E-RS scoring algorithm would be needed for use in spirometric ACO.

#### Reliability

With the factor structure in place, internal consistency (the extent to which individual items within an instrument or its domains are inter-related) of the E-RS was evaluated using Cronbach’s alpha at Day − 1 and Day 28. Values > 0.70 were considered acceptable for aggregate data. To assess the reproducibility of scores over time, test-retest reliability of the E-RS scores and wheeze item were analyzed using intra-class correlations (ICC) and paired *t*-tests between Days − 2 and − 1 and between Days − 7 and − 6. ICC values > 0.70 were considered acceptable. [28] Test-retest analyses were also conducted for patients with stable PEF over 2 days (change in daily morning PEF of < 15%).

#### Construct validity

To determine if the E-RS and wheeze item measure the constructs they were designed to measure, correlations between these instruments and conceptually-related measures were assessed. Using scores averaged across the baseline week and the final treatment week, Spearman’s correlation coefficients were calculated between E-RS or wheeze scores and the following criterion variables: SGRQ Total and domain scores, average daily rescue medication use, average morning PEF, average FEV_1_ from patient-initiated spirometry and clinic-collected FEV_1_% predicted. Moderate–strong correlations (r > 0.4) were expected for the SGRQ scores, while weak correlations were expected for pulmonary function, consistent with previous findings. [19, 20]

Known-groups validity was tested using an analysis of variance to evaluate the relationship between the mean of daily E-RS and wheeze item scores during the baseline week and categories commonly used in the criterion variables: exacerbation history (≥1 or 0) prior to Day − 28, FEV_1_% predicted (GOLD guidelines: ≥80%, 50–80, < 50%) at Day − 28 and Day 1, mMRC dyspnea status (0–1, 2, 3–4) at Day 1 and primary diagnosis (asthma or COPD) at Day − 28.

#### Responsiveness

The responsiveness of the E-RS and wheeze item, which refers to the ability of these measures to detect change over time, was examined using an analysis of covariance among patients considered to be responders from the baseline week to the final treatment week. Patients were assigned to responder groups based on their PGIC score (better/much better, no change/slightly better, slightly worse/worse), and changes in SGRQ Total scores (responder thresholds: <− 4 [better], − 4 to 4 [no change] and > 4 [worse]). [29] The E-RS and wheeze item were considered responsive if scores improved or declined in these responder groups (results in the no-change group provided evidence of reproducibility). To understand the magnitude of change, effect sizes were calculated; effect sizes of 0.20, 0.5 and 0.8 were interpreted as small, moderate and large, respectively. [28]

#### Within-patient change thresholds

An anchor-based approach was used to identify the threshold of within-patient change considered meaningful for E-RS (Total and domain) and wheeze item scores. Meaningful score improvements were identified by a PGIC score of 1 (slightly better) or a decrease (improvement) in SGRQ Total score of 4.0–5.0 points inclusive. E-RS and wheeze scores representing meaningful deterioration were defined by a PGIC score of − 1 (slightly worse) or an increase in SGRQ Total score of 4.0–5.0 points inclusive.

## Results

### Patient population

A total of 338 patients were randomized to receive trial treatment, of whom 97% completed the trial. Most patients were ≥ 40 years of age (95%), white (98%), 53% were male, and 54 and 46% had a primary diagnosis (according to patient medical records) of asthma or COPD, respectively (Table 1). A total of 227 (67%) and 273 (81%) of patients had a diagnosis by a HCP of asthma and COPD, respectively. All patients met the spirometric ACO criteria at baseline. Spirometric analyses by primary diagnosis are included in Additional file 1: Table S1. Most (63%) patients were current or former smokers, with an overall mean of 24.4 pack-years at screening. For patients with a primary diagnosis of asthma or COPD, 56 and 15% of patients indicated that they had never smoked and 45 and 85% were current or former smokers, respectively (data not shown). The mean (standard deviation [SD]) baseline E-RS Total (RS-Total) and SGRQ Total scores were 10.7 (6.1) and 44.0 (15.3) respectively, across treatment groups.

**Table 1:** Baseline characteristics^a^

	Overall population (*N* = 338)
Age (years), mean (SD)	57.5 (10.6)
≥40 years, n (%)	321 (95)
< 40 years, n (%)	17 (5)
Male, n (%)	178 (53)
Ethnicity n, (%)	
Hispanic/Latino	13 (4)
Not Hispanic/Latino	325 (96)
Body mass index (kg/m^2^), mean (SD)	27.9 (5.0)
Primary diagnosis, n (%)	
Asthma	183 (54)
COPD	155 (46)
Smoking status, n (%)	
Never	125 (37)^b^
Former	129 (38)
Current	84 (25)
Pack years^c^, mean (SD)	24.4 (21.8)
Pre-bronchodilator FEV_1_ (L), mean (SD)	1.57 (0.5)
Pre-bronchodilator FEV_1_ (% predicted), mean (SD)	51.0 (8.2)
Post-bronchodilator FEV_1_ (% predicted), mean (SD)	64.8 (7.7)
Pre-bronchodilator FEV_1_/FVC (%), mean (SD)	50.5 (8.1)
Post-bronchodilator FEV_1_/FVC, (%), mean (SD)	55.5 (7.8)
FEV_1_ reversibility (mL), mean (S^d^)	423.0 (184.0)
E-RS Total score (range: 0–40), mean (SD)	10.7 (6.1)
SGRQ Total score (range: 0–100), mean (SD)	44.0 (15.3)
SGRQ Activity score (range: 0–100), mean (SD)	53.7 (18.5)
SGRQ Impacts score (range: 0–100), mean (SD)	33.7 (17.0)
SGRQ Symptoms score (range: 0–100), mean (SD)	60.0 (19.4)
mMRC dyspnea scale score (range: 0–4), mean (SD)	1.8 (0.7)
0–1, n (%)	114 (34)
≥ 2, n (%)	224 (66)
Blood eosinophils, n (%)	
< 0.15 GI/L	135 (42)
≥ 0.15 GI/L	183 (58)
Wheeze score (range: 0–4), mean (SD)	0.9 (0.8)

^a^Data adapted from Lee et al., 2017, *Respir Med*, published by Elsevier. This is an open access article under the terms of the Creative Commons Attribution-Non Commercial-No Derivatives 4.0 International License (10.1016/j.rmed.2017.08.013) [24]; ^b^Of whom, 102 and 23 patients had asthma or COPD as a primary diagnosis, respectively; ^c^*n* = 213. ^d^TT: FEV_1_ reversibility was < 400 mL for 185 patients (55%) and ≥ 400 mL for 153 patients (45%)

*COPD* chronic obstructive pulmonary disease, *E-RS* Evaluating Respiratory Symptoms in COPD, *FEV*_*1*_ forced expiratory volume in 1 s, *FVC* forced vital capacity, *mMRC* Modified Medical Research Council, *SD* standard deviation, *SGRQ* St George’s Respiratory Questionnaire

### Item analysis

#### Inter-item correlation

Descriptive statistics for the E-RS and wheeze item are summarized in Additional file 1: Table S2. In the overall sample, correlations between items within the same domain were stronger (0.4–1.0) than those across different domains (0.2–0.6) at Day − 1 (Table 2). There was a moderate correlation (all > 0.5 and < 0.7) between ‘chest symptoms’ (items 5 and 6 [Chest domain]) and ‘chest congested’ (item 1 [Cough and Sputum domain]) and ‘breathless’ (item 7 [Breathlessness domain]).

**Table 2:** Inter-item correlations^a^ for E-RS items and wheeze item at Day − 1 for the Overall population (*N* = 328)

Variable	Chest congested	Cough	Mucus when coughing	Difficulty with mucus	Chest discomfort	Chest tight	Breathless	How breathless	Short of breath with personal care	Short of breath with indoor	Short of breath with outdoor	Wheeze
1. Chest congested	1	0.5	0.5	0.6	0.6	0.6	0.5	0.5	0.5	0.5	0.5	0.5
2. Cough	0.5	1	0.6	0.5	0.5	0.4	0.4	0.3	0.3	0.4	0.4	0.4
3. Mucus when coughing	0.5	0.6	1	0.5	0.3	0.2	0.2	0.2	0.3	0.3	0.2	0.3
4. Difficulty with mucus	0.6	0.5	0.5	1	0.4	0.4	0.4	0.4	0.4	0.5	0.4	0.5
5. Chest discomfort	0.6	0.5	0.3	0.4	1	0.8	0.6	0.5	0.5	0.5	0.5	0.5
6. Chest tight	0.6	0.4	0.2	0.4	0.8	1	0.6	0.5	0.5	0.5	0.5	0.5
7. Breathless	0.5	0.4	0.2	0.4	0.6	0.6	1	0.7	0.5	0.6	0.7	0.5
8. How breathless	0.5	0.3	0.2	0.4	0.5	0.5	0.7	1	0.5	0.5	0.6	0.5
9. Short of breath with personal care	0.5	0.3	0.3	0.4	0.5	0.5	0.5	0.5	1	0.7	0.6	0.4
10. Short of breath (indoor)	0.5	0.4	0.3	0.5	0.5	0.5	0.6	0.5	0.7	1	0.7	0.4
11. Short of breath (outdoor)	0.5	0.4	0.2	0.4	0.5	0.5	0.7	0.6	0.6	0.7	1	0.5
Wheeze	0.5	0.4	0.3	0.5	0.5	0.5	0.5	0.5	0.4	0.4	0.5	1

^a^Data were analyzed using Spearman’s correlation

Correlations > 0.40 and > 0.70 were defined as moderate and strong, respectively

*E-RS* Evaluating Respiratory Symptoms in chronic obstructive pulmonary disease

### Factor analysis

**Fig. 1:**
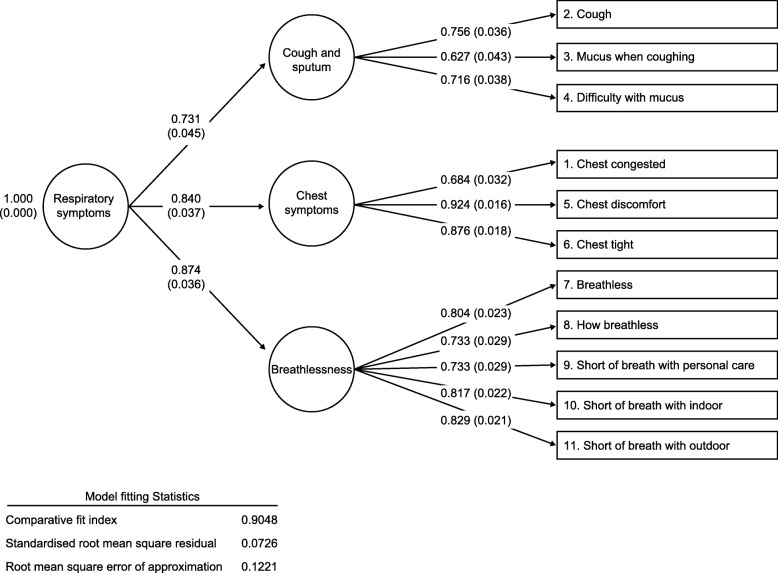
Confirmatory factor analysis of the E-RS at Day − 1 for the overall population (N = 328). Data are displayed as the correlation coefficient r (p-value). Factor loadings > 0.40 were considered acceptable

In the CFA, the 11 E-RS items loaded clearly onto the domains of Cough and Sputum, Chest Symptoms and Breathlessness (Fig. 1). Model fit was strong (comparative fit index: 0.9, root mean square error of approximation: 0.1). In the exploratory factor analysis (EFA; Additional file 1: Table S3), the wheeze item did not load onto any of the E-RS domains (promax rotation factor loading across domains: < 0.37). The factor analysis results confirmed the factor structure of the E-RS, excluding wheeze, and including a Total score and three domain scores, for use in patients with spirometric ACO. Thus, the E-RS structure was retained for tests of reliability, validity and responsiveness and the wheeze item was analyzed separately.

### Reliability

RS-Total and domain scores were internally consistent at Day − 1 (Cronbach’s alpha: 0.7–0.9) and Day 28 (Cronbach’s alpha: 0.8–0.9). All scores exceeded the 0.7 threshold at Day − 1 (Table 3) and Day 28 (data not shown).

**Table 3:** Internal consistency and test-retest reliability of E-RS Total and domain scores and wheeze

			Internal consistency	Test-retest reliability		
			Day − 1 (*N* = 328)	Day − 2 to Day − 1^a^		
	Scale	No. items	Cronbach’s alpha (raw)	Difference in mean (SD) score between days	*p*-value	ICC (95% CI)
All	RS-Total	11	0.91	0.21	0.2983	0.83 (0.79–0.86)
	RS-Cough and Sputum	4	0.71	0.07	0.2686	0.78 (0.74–0.82)
	RS-Chest Symptoms	2	0.86	0.03	0.6776	0.73 (0.67–0.78)
	RS-Breathlessness	5	0.89	0.10	0.3795	0.80 (0.76–0.84)
	Wheeze	1	–	0.03	0.3411	0.75 (0.70–0.80)
Morning PEF change < 15%	RS-Total	11	–	0.30	0.2492	0.82 (0.77–0.86)
	RS-Cough and Sputum	4	–	0.11	0.1877	0.77 (0.70–0.82)
	RS-Chest Symptoms	2	–	0.04	0.6643	0.72 (0.65–0.78)
	RS-Breathlessness	5	–	0.14	0.3276	0.81 (0.76–0.85)
	Wheeze	1	–	0.06	0.1216	0.78 (0.71–0.82)

^a^For Days −2 to −1, *n* = 321 for all patients and *n* = 202 for morning PEF change < 15%

*CI* confidence intervals, *E-RS* Evaluating Respiratory Symptoms in chronic obstructive pulmonary disease, *ICC* intra-class correlations, *PEF* peak expiratory flow, *RS* respiratory symptom, *SD* standard deviation

Over the Day − 2 to Day − 1 test-retest, E-RS and wheeze item scores were reproducible, with all ICC scores reaching the 0.7 threshold (Table 3). There were no significant differences between Day − 2 and Day − 1 tests for E-RS scores or wheeze item scores (*p* = 0.3–0.7). From Day − 7 to Day − 6, E-RS scores were reproducible (*p* = 0.4–0.9), although the ICC score for the wheeze item did not reach 0.7 (0.67; *p* = 0.56; Additional file 1: Table S4). E-RS scores were also reproducible in patients who exhibited < 15% change from the previous day in morning PEF for both test-retests (Table 3 and Additional file 1: Table S4).

### Construct validity

RS-Total and RS-Breathlessness scores were correlated with SGRQ Total and Activity domain scores (r = 0.43 to 0.48) during the baseline week (Table 4). Correlations between the RS-Total and RS-Breathlessness scores and the SGRQ Impacts and Symptoms domain scores were lower (r = 0.35 to 0.39) (Table 4). The correlations between RS-Total and domain scores and pulmonary function variables were low (morning PEF: r = − 0.19 to − 0.33; FEV_1_: r = − 0.06 to − 0.24; FEV_1_% predicted: r = − 0.05 to − 0.22). For the wheeze item during the baseline week, correlation with the SGRQ Symptom domain score was moderate (r = 0.41) and correlations with pulmonary function variables were weak (morning PEF: -0.12; FEV_1_: r = 0.01; FEV_1_% predicted: r = − 0.11). Correlations during the final treatment week are shown in Table 4.

**Table 4:** Construct validity: E-RS and wheeze Spearman correlations with SGRQ and clinical assessments

	Baseline week (Day − 7 to Day − 1)								Final treatment week (Day 21 to Day 28)							
	SGRQ: Total	SGRQ: Activity	SGRQ: Impacts	SGRQ: Symptoms	Rescue medication use	Morning PEF	FEV_1_	FEV_1_% predicted	SGRQ: Total	SGRQ: Activity	SGRQ: Impacts	SGRQ: Symptoms	Rescue medication use	Morning PEF	FEV_1_	FEV_1_% predicted
N	326	328	329	327	336	338	338	338	321	324	325	325	326	327	327	327
RS-Total	**0.46**	**0.43**	0.36	0.39	0.33	−0.30	−0.18	−0.17	**0.52**	**0.54**	0.40	**0.43**	0.40	−0.27	− 0.18	− 0.17
RS-Cough and Sputum	0.32	0.29	0.22	0.35	0.26	−0.23	−0.11	− 0.11	0.38	0.36	0.29	**0.44**	0.34	− 0.16	− 0.11	− 0.10
RS-Chest Symptoms	0.37	0.32	0.3	0.33	0.29	− 0.19	−0.06	− 0.05	**0.45**	**0.43**	0.37	0.39	0.32	−0.18	− 0.09	− 0.08
RS-Breathlessness	**0.48**	**0.48**	0.38	0.35	0.33	−0.33	−0.24	− 0.22	**0.53**	**0.59**	**0.42**	0.38	0.40	−0.31	−0.23	− 0.23
Wheeze	0.38	0.33	0.29	**0.41**	0.33	−0.12	0.01	−0.11	**0.42**	0.38	0.34	**0.43**	0.32	−0.12	−0.04	− 0.06

Weak, moderate and strong correlations were defined as r ≤ 0.4 (unmarked), r > 0.4 (bold) and r > 0.7 (none present in this table), respectively

*E-RS* Evaluating Respiratory Symptoms in chronic obstructive pulmonary disease, *FEV*_*1*_ forced expiratory volume in 1 s, *PEF* peak expiratory flow, *RS* respiratory symptoms, *SGRQ* St George’s Respiratory Questionnaire

**Fig. 2:**
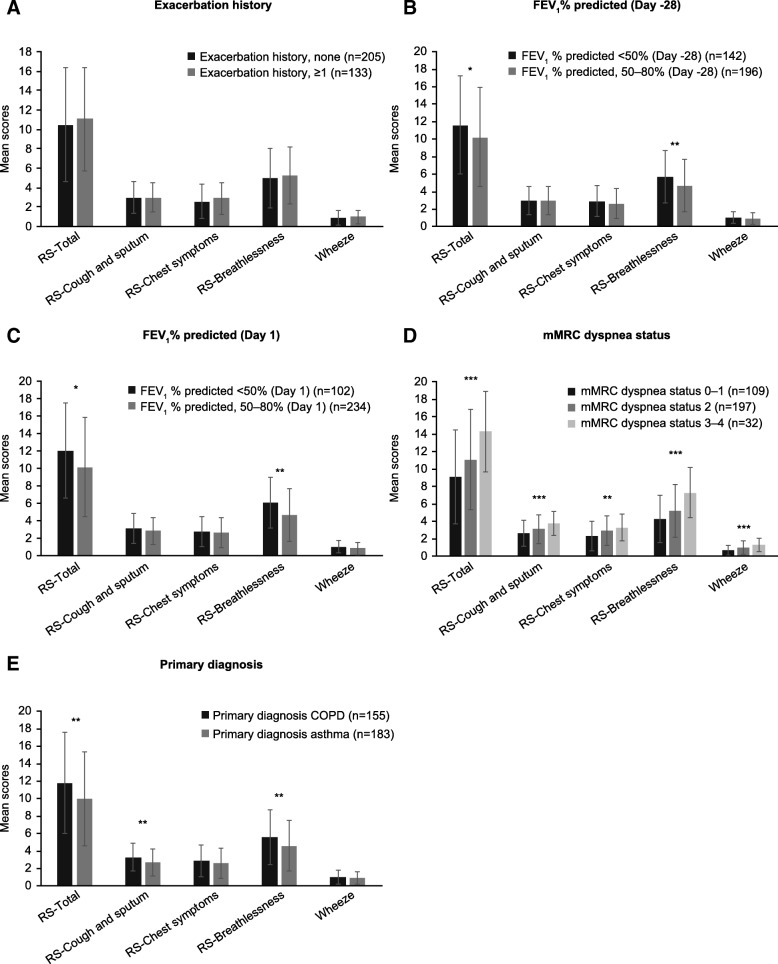
Mean (SD) known groups validity of E-RS Total and domain scores and wheeze item at the baseline week. The baseline week was defined as the last week of run-in (Day − 7 to Day − 1). *p < 0.05; **p < 0.01; ***p < 0.001; Error bars show standard deviations. COPD, chronic obstructive pulmonary disease; E-RS, Evaluating Respiratory Symptoms in COPD; FEV_1_, forced expiratory volume in 1 s; mMRC, Modified Medical Research Council; SD, standard deviation

In the known-groups validity analysis, RS-Total scores during the baseline week differentiated between patients grouped according to FEV_1_% predicted at Day − 28 (*p* = 0.0323) and Day 1 (*p* = 0.0154), mMRC dyspnea status (*p* < 0.0001) and primary diagnosis of asthma versus COPD (*p* = 0.0033), but not exacerbation history (*p* = 0.3378; Fig. 2). The wheeze item during the baseline week differentiated between patients grouped according to mMRC dyspnea status (*p* = 0.0002) but not exacerbation history (*p* = 0.081), FEV_1_% predicted at Day − 28 (*p* = 0.1986) or Day 1 (*p* = 0.147) or primary diagnosis (*p* = 0.45).

### Responsiveness

**Fig. 3:**
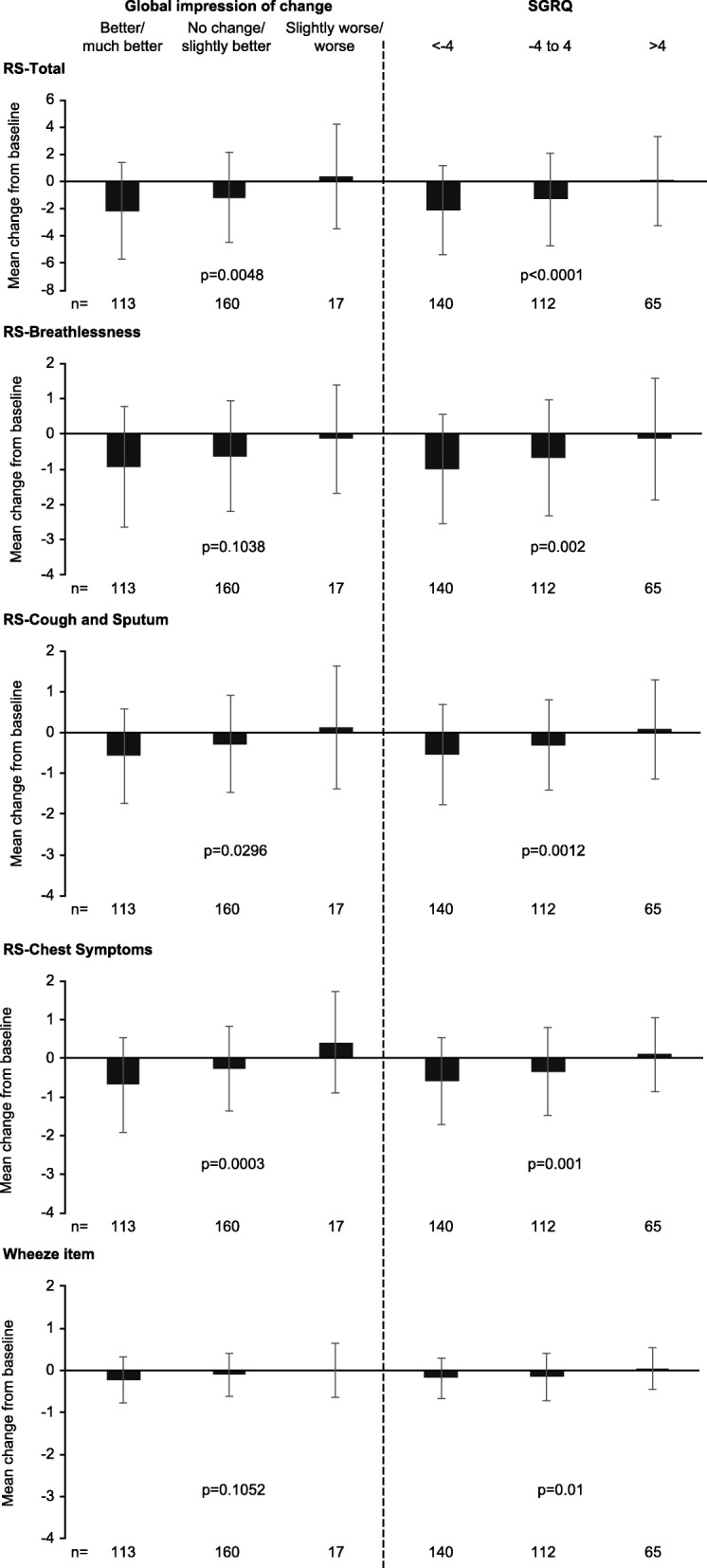
Responsiveness for E-RS scores and wheeze item (mean change from baseline week to final week). The baseline week was defined as the last week of run-in (Day − 7 to Day − 1). The final week was defined as the final week of the 4-week treatment period (Day 21 to Day 28). E-RS, Evaluating Respiratory Symptoms in chronic obstructive pulmonary disease; SGRQ, St George’s Respiratory Questionnaire. Of the 160 patients categorized as no change/slightly better, 31 were categorized as no change and 129 as slightly better. Of the 17 patients categorized as slightly worse/worse, 14 were categorized as slightly worse and 3 as worse

For patients rated as better/much better from baseline to Day 28 on the PGIC scale for lung condition (overall disease), mean (SD) change from baseline to final week in RS-Total was − 2.2 (3.6), with an effect size of − 0.4, representing a small to moderate improvement (Fig. 3). Similarly, for patients with a SGRQ Total score improvement from baseline, mean (SD) change in RS-Total from baseline to final week was − 2.1 (3.3) with an effect size of − 0.4. Mean (SD) change in RS-Total for patients with relatively stable disease, as indicated by no change on the PGIC scale or − 4 to 4 on the SGRQ scale, was − 1.2 (3.55) and − 1.3 (3.43), respectively, each with small effect sizes of − 0.2. Responsiveness of E-RS domain scores is shown in Fig. 3.

For the wheeze item, mean (SD) change in wheeze scores from baseline to final week was − 0.22 (0.54) for patients rated as better/much better on the PGIC scale and − 0.18 (0.48) for patients with an improvement on the SGRQ scale, both with an effect size of − 0.3 (Fig. 3). In stable patients, according to PGIC and SGRQ scales, the mean (SD) change in wheeze scores was − 0.1 (0.52) and − 0.2 (0.56), with effect sizes of − 0.1 and − 0.2, respectively.

### Within-patient change thresholds

Patients reporting slightly better lung condition (overall disease) on the PGIC (1 point) in the final week relative to baseline had a mean (SD) change in RS-Total score of − 1.4 (3.4), while those with a meaningful improvement in health status based on a change in SGRQ Total score of 4 to 5 points had a mean change score in RS-Total of − 2.7 (3.4) (Table 5). RS-Cough and Sputum, RS-Chest Symptoms and RS-Breathlessness values in patients rated as slightly better (*n* = 129) on the PGIC scale were − 0.3 (1.2), − 0.4 (1.1) and − 0.7 (1.6), respectively, while those with health status improvements (*n* = 8) as assessed by SGRQ had values of − 0.7 (0.6), − 0.8 (1.4), and − 1.2 (1.6), respectively (Table 5). Values for the wheeze item are shown in Table 5.

**Table 5:** Within-patient change threshold analyses: Mean change from baseline by anchor-based methods for E-RS Total and domain scores and wheeze item

Item	Subgroup	Mean change from baseline to final week^a^ (SD)
RS-Total	Global impression of change: slightly better (*n* = 129)	−1.4 (3.4)
	Global impression of change: slightly worse (*n* = 14)	0.2 (3.7)
	SGRQ improvement: decrease 4 to 5 points (*n* = 8)	−2.7 (3.4)
	SGRQ deterioration: increase 4 to 5 points (*n* = 11)	−1.4 (2.9)
RS-Cough and Sputum	Global impression of change: slightly better (*n* = 129)	−0.3 (1.2)
	Global impression of change: slightly worse (*n* = 14)	0.0 (1.4)
	SGRQ improvement: decrease 4 to 5 points (*n* = 8)	−0.7 (0.6)
	SGRQ deterioration: increase 4 to 5 points (*n* = 11)	0.3 (0.6)
RS-Chest Symptoms	Global impression of change: slightly better (*n* = 129)	−0.4 (1.1)
	Global impression of change: slightly worse (*n* = 14)	0.5 (1.4)
	SGRQ improvement: decrease 4 to 5 points (*n* = 8)	−0.8 (1.4)
	SGRQ deterioration: increase 4 to 5 points (*n* = 11)	−0.6 (1.2)
RS-Breathlessness	Global impression of change: slightly better (*n* = 129)	−0.7 (1.6)
	Global impression of change: slightly worse (*n* = 14)	−0.3 (1.4)
	SGRQ improvement: decrease 4 to 5 points (*n* = 8)	−1.2 (1.6)
	SGRQ deterioration: increase 4 to 5 points (*n* = 11)	−0.5 (1.4)
Wheeze	Global impression of change: slightly better (*n* = 129)	−0.1 (0.5)
	Global impression of change: slightly worse (*n* = 14)	−0.1 (0.6)
	SGRQ improvement: decrease 4 to 5 points (*n* = 8)	−0.2 (0.6)
	SGRQ deterioration: increase 4 to 5 points (*n* = 11)	−0.2 (0.5)

^a^The baseline week was defined as the last week of run-in (Day −7 to Day −1). The final week was defined as the final week of the 4-week treatment period (Day 21 to Day 28)

*E-RS* Evaluating Respiratory Symptoms in chronic obstructive pulmonary disease, *SD* standard deviation, *SGRQ* St George’s Respiratory Questionnaire

### Impact of primary diagnosis

The psychometric properties of the E-RS and wheeze item were consistent between patients with a primary diagnosis of asthma and COPD (Additional file 1: Tables S5–S13). Further details are described in the Additional File 1.

## Discussion

These post hoc analyses indicate that E-RS scores were reliable, valid and responsive in patients with spirometric ACO (fixed airflow obstruction and reversibility to salbutamol), including patients whose underlying respiratory disease was classified as primarily asthma or COPD who were enrolled in a clinical trial to test the effects of pharmaceutical agents on change from baseline in clinic trough (pre-dose) FEV_1_. [24] The higher-order factor structure of the E-RS in the present study excluded wheeze and supported three domain and Total scale scores, consistent with the structure of the E-RS in COPD. [19, 20] Total and domain scores showed high levels of internal consistency, similar to those reported in patients with COPD (0.70–0.90), indicating a high degree of precision with low measurement error. [19, 20] Although the strength and pattern of relationships observed between E-RS scores and both SGRQ scores and pulmonary function variables supported the validity of the instrument in this sample, the correlations between the E-RS and SGRQ scores were not as strong as those shown in COPD. [20] For example, the correlation between the E-RS and SGRQ Total scores was 0.75 in COPD compared with 0.46 in this study. [20] The weak correlations between RS-Total and SGRQ domain scores and pulmonary function variables in the present study were consistent with those reported in COPD. [20, 21] Meaningful improvements in RS-Total score were determined based on a PGIC score of 1 (slightly better) or a decrease (improvement) in SGRQ Total score of greater than − 4 points. As these corresponded with mean changes in RS-Total scores of − 1.4 and − 2.7, respectively, scores greater than − 1.4 may be used as a starting point for interpreting change scores in clinical trials evaluating the effect of treatment on respiratory symptoms in patients with spirometric ACO. This estimate is smaller than the estimate of − 2.0 in COPD. [19] Patient numbers for some of the subgroups assessed in the within-patient change threshold analyses were low (*n* = 8–14), and therefore, care should be taken when drawing conclusions from these data.

Findings for the wheeze item were less clear. The item did not correlate with E-RS items, nor did it fit into the factor structure of the E-RS. The wheeze item did correlate with SGRQ scores but known-groups validity was poor and limited to differentiating only mMRC dyspnea levels. Responsiveness analyses demonstrated limited sensitivity of the wheeze item to change, which failed to show a difference between groups stratified by PGIC or change in SGRQ. Although patients with spirometric ACO have described ‘wheeze’ as part of their symptom experience, their descriptions of its precise nature vary, [12] making standardized assessment of wheeze from a patient perspective difficult. This variation is consistent with qualitative descriptions of wheeze in patients with COPD. [20] Wheeze may be best reclassified as a sign of disease and assessed by clinicians through auditory evaluation (lung sounds), rather than patient self-report. Alternatively, self-report of wheeze with varied terminology could be explored in future studies.

In this study, patients reported a mean (SD) RS-Total score of 10.7 (6.1) at Day − 1, which is lower than the mean scores (11.0–18.2) reported in studies of mild to very severe COPD across different trial populations. [19, 30–32] This suggests that the patients in this study were less symptomatic than typical patients with stable COPD. [19] In contrast, the health status of this sample, as assessed by SGRQ (mean [SD] Total scores: 44.0 [15.3]), was comparable to patients with mild to very severe COPD (mean SGRQ: 47.6, range: 29.2–55.2 across subgroups by symptom), [14] moderate to severe COPD (mean SGRQ: 47.8–49.6 across treatment groups), [33] severe COPD (mean SGRQ: 53.5–54.8 across treatment groups) [34] and severe asthma (41.2). [35] This suggests that factors other than symptoms may be limiting health status in patients with spirometric ACO.

Limitations of this study include that the standardized diagnostic criteria used for ACO are debatable. [9, 10] Therefore, the study sample may not fulfill all of the elements contained in the recently proposed ACO criteria. [10] For example, a recent consensus statement suggests patients with ACO usually present with persistent airflow limitation, at least 10 pack-years of smoking, and documentation of asthma before 40 years of age OR a bronchodilator response of > 400 mL (major criteria). [10] Minor criteria include history of atopy or allergic rhinitis, a bronchodilator response of FEV_1_ ≥ 200 mL and 12% and a peripheral blood eosinophil count of ≥300 cells per μL. [10] In this study, although most patients were current or former smokers, 37% of patients had never smoked, of whom, 56 and 15% had a primary diagnosis of asthma and COPD, respectively. In addition, only 46% of patients demonstrated FEV_1_ reversibility of ≥400 mL. However, consistent with the recent consensus definition of ACO, most patients had received a diagnosis of asthma (primary diagnosis: 54%, diagnosis by a HCP: 67%), most were ≥ 40 years of age (95%), and all exhibited persistent airflow limitation (post-bronchodilator FEV_1_/forced vital capacity < 0.70) and a post-bronchodilator increase in FEV_1_ of ≥12% and ≥ 200 mL. This indicates that the sample generally represents patients with ACO and ACO-like illness sufficient to test the performance properties of the E-RS as a measure of respiratory symptoms in studies of ACO. Of note, smoking is not included as standard across all definitions of ACO, [36, 37] and although smoke and/or biomass exposure are compatible with an ACO diagnosis, the condition is present in up to 60% of non-smokers when diagnosis is based on lung function criteria [38], supporting the relevance of the population reported in this study. These results are also the first to suggest a suitable tool for measuring symptoms in spirometric ACO regardless of primary diagnosis. While we acknowledge that our spirometric ACO population may differ slightly from other ACO populations in terms of its clinical characteristics, we stress that none of the features of asthma or COPD is pathognomonic, as discussed by Bateman, et al., [38] and our results are relevant in a spirometric ACO population as defined herein. As our analysis included patients from a single clinical trial and was defined by criteria based on spirometric outcomes, these results may not be generalizable to all patients with ACO.

## Conclusion

Results of this study suggest that the E-RS:COPD may be useful for quantifying treatment effects on respiratory symptoms in clinical trials of patients with spirometric ACO. Further study in patients with ACO is warranted as the characteristic features of this clinical population become more clearly defined.

## Additional file

Additional file 1: Includes additional text describing the patient sample, the descriptive statistics for the E-RS and wheeze item, and the results of the inter-item correlations, factor analysis and known-groups validity by primary diagnosis. Descriptive statistics for the E-RS and wheeze item scores for Day − 1 and Day 28 and the EFA at Day − 1 for E-RS with and without the wheeze item in the overall population and by primary diagnosis of asthma and COPD are also described. (DOCX 87 kb)

## Abbreviations

ACO: Asthma-COPD overlap
CFA: Confirmatory factor analysis
COPD: Chronic obstructive pulmonary disease
EFA: Exploratory factor analysis
E-RS:COPD: Evaluating Respiratory Symptoms in COPD
EXACT: Exacerbations of Chronic Pulmonary Disease Tool
FEV_1_: Forced expiratory volume in one second
GINA: Global Initiative for Asthma
GOLD: Global Initiative for Chronic Obstructive Lung Disease
ICC: Intra-class correlations
mMRC: modified Medical Research Council
PEF: Peak expiratory flow
PGIC: Patient Global Impression of Change
PRO: Patient-reported outcome
SD: Standard deviation
SGRQ: St George’s Respiratory Questionnaire
