# Supplementary material for: Reliability, validity and responsiveness of E-RS:COPD in patients with spirometric asthma-COPD overlap
Source: Respir Res. 2019 May 31;20:107. doi: 10.1186/s12931-019-1070-6 (PMC6545030; doi:10.1186/s12931-019-1070-6)
Supplement: Supplementary file 1 — Includes additional text describing the patient sample, the descriptive statistics for the E-RS and wheeze item, and the results of the inter-item correlations, factor analysis and known-groups validity by primary diagnosis. Descriptive statistics for the E-RS and wheeze item scores for Day − 1 and Day 28 and the EFA at Day − 1 for E-RS with and without the wheeze item in the overall population and by primary diagnosis of asthma and COPD are also described. (DOCX 87 kb) [file 12931_2019_1070_MOESM1_ESM.docx]

# SUPPLEMENTARY MATERIALS

# Methods

## Patient sample

Patients were ≥18 years of age with chronic obstructive pulmonary disease (COPD; American Thoracic Society/European Respiratory Society definition)[1] and an asthmatic component evidenced by all three of the following criteria: 1) a post-bronchodilator morning forced expiratory volume in 1 second (FEV_1_) % predicted of ≥50% and ≤80% and a pre- and post-bronchodilator FEV_1_/forced vital capacity ratio of <0.7; 2) reversible disease defined as a ≥12% and ≥200 mL increase in FEV_1_ in 20–60 minutes following four inhalations of salbutamol aerosol; 3) receipt of inhaled corticosteroid-containing therapy for ≥12 weeks with a stable dose during the 4 weeks before the run-in period. Further inclusion and exclusion criteria have been reported previously.[2] Patients were assigned a primary diagnosis of either asthma or COPD by the physician at the beginning of the run-in period, based on medical history and clinical judgement following patient interview. No standardized formal diagnosis criteria were applied.

# Results

Descriptive statistics for the Evaluating Respiratory Symptoms in COPD (E-RS:COPD) and wheeze item data are summarized in Table S1. Respiratory symptom (RS)-Total scores improved from 10.7 to 8.7 from Day -1 to Day 28, meeting the ≥2.0-point reduction criteria used to define responders in COPD. The wheeze item scores also improved from 0.9 to 0.7. Item responses and scale ranges had minimal ceiling effects at Day -1 and Day 28.

## Impact of primary diagnosis

### Inter-item correlation

Descriptive statistics for E-RS and wheeze item data by primary diagnosis are summarized in Table S3. In patients with asthma and COPD, correlations between items within the same domain were stronger (0.4–1.0) than those across different domains (0.2–0.6) at Day -1 (Tables S4 and S5) and 28 (data not shown).

Similar to the overall population, there was a strong correlation between chest symptoms (items 5 and 6 [Chest domain]) and chest congested (item 1 [Cough and Sputum domain]) and breathless (item 7 [Breathlessness domain]) in patients with asthma. However, in patients with COPD, there was no strong correlation between the chest congested and breathless items.

### Factor analysis

In patients with a primary diagnosis of asthma or COPD, the confirmatory factor analysis (CFA) demonstrated that the individual items of the E-RS loaded clearly onto the three domains of Breathlessness, Cough and Sputum, and Chest and Symptoms (Table S6 and S7). The CFA provided a strong model fit in both patient groups at Day -1 (comparative fit index: 0.9, root mean square error of approximation: 0.1). In both patient populations, the exploratory factor analysis (EFA) demonstrated that the wheeze item did not fit within the structure of the E-RS (promax rotation factor loading: ≤0.35; Tables S8 and S9).

### Known-groups validity

In patients with asthma, RS-Total scores during the baseline week differentiated between patients grouped according to FEV_1_% predicted at Day -28 (p=0.0407) but not at Day 1 (p=0.0909). RS-Breathlessness scores differentiated between these patient groups at Day -28 (p=0.0086) and also at Day 1 (p=0.0062). The RS-Total, RS-Breathlessness, RS-Cough and Sputum scores and the wheeze item all differentiated between patients grouped according to modified Medical Research Council (mMRC) dyspnea status, with p‑values ranging from p=0.0122 to p=0.0316. The E-RS total and domain scores and the wheeze item did not differentiate between patients grouped according to exacerbation history (p=0.4996–0.9545; Table S10).

In patients with COPD, only the RS-Breathlessness score differentiated between patients grouped according to FEV_1_ % predicted at Day 1 (p=0.0355), and the RS-Total, RS-Breathlessness, RS-Cough and Sputum scores and wheeze item differentiated between patients grouped according to mMRC dyspnea status (p=0.0016–0.0346). The E-RS Total and domain scores and the wheeze item did not differentiate between patients grouped according to exacerbation history (p=0.0748–0.7144) or FEV_1_ % predicted at Day -28 (p=0.1916–0.9415; Table S11).

# Table S1 Spirometric characteristics by primary diagnosis (asthma: n=183, COPD: n=155) at Visit 1*

| **Baseline characteristic** | **Asthma (n=183)** | **COPD (n=155)** |
| --- | --- | --- |
| Pre-bronchodilator FEV_1_ (L), mean (SD) | 1.7 (0.5) | 1.5 (0.4) |
| Post-bronchodilator FEV_1_ (L), mean (SD) | 2.1 (0.6) | 1.9 (0.4) |
| Pre-bronchodilator FEV_1_ (% predicted), mean (SD) | 51.6 (8.2) | 50.2 (8.2) |
| Post-bronchodilator FEV_1_ (% predicted), mean (SD) | 66.1 (7.8) | 63.4 (7.2) |
| Pre-bronchodilator FEV_1_/FVC (%), mean (SD) | 51.8 (7.5) | 48.9 (8.4) |
| Post-bronchodilator FEV_1_/FVC, (%), mean (SD) | 57.2 (7.1) | 53.5 (8.0) |
| FEV_1_ reversibility (mL), mean (SD) | 458.2 (199.9)^†^ | 381.5 (153.8)^‡^ |

*Data adapted from Lee et al, 2017, *Respir Med*, published by Elsevier. This is an open access article under the terms of the Creative Commons Attribution‐Non Commercial-No Derivatives 4.0 International License ([https://doi.org/10.1016/j.rmed.2017.08.013)[2](https://doi.org/10.1016/j.rmed.2017.08.013)%5b2)]

^†^FEV_1_ reversibility was <400 mL for 85 (46%) patients and ≥400 mL for 98 (54%) patients; ^‡^FEV_1_ reversibility was <400 mL for 100 (65%) patients and ≥400 mL for 55 (35%) patients.

COPD, chronic obstructive pulmonary disease; FEV_1_, forced expiratory volume in 1 second; FVC, forced vital capacity; SD, standard deviation.

# Table S2. Descriptive statistics for E-RS domain and Total scores and wheeze item score for Day -1 (N=328) and Day 28 (N=261) (overall population)

|  |  | **Day -1** | | | | **Day 28** | | | |
| --- | --- | --- | --- | --- | --- | --- | --- | --- | --- |
|  | **Item*** | **Mean (SD)** | **Observed range** | **Floor (%)** | **Ceiling (%)** | **Mean (SD)** | **Observed range** | **Floor (%)** | **Ceiling (%)** |
| **E-RS Cough and Sputum domain** | **1. Chest congested** | 0.9 (0.7) | 0–3 | 29.6 | 0 | 0.8 (0.7) | 0–3 | 39.9 | 0 |
|  | **2. Cough** | 1.2 (0.8) | 0–4 | 18.3 | 0.9 | 1.0 (0.8) | 0–3 | 28.4 | 0 |
|  | **3. Mucus when coughing** | 0.8 (0.5) | 0–2 | 24.4 | 0 | 0.7 (0.5) | 0–2 | 34.9 | 0 |
|  | **4. Difficulty with mucus** | 1.0 (0.9) | 0–4 | 29.6 | 0.9 | 0.8 (0.8) | 0–4 | 41.4 | 0.4 |
| **E-RS Chest Symptoms domain** | **5. Chest discomfort** | 0.9 (0.8) | 0–4 | 30.8 | 0.3 | 0.7 (0.7) | 0–2 | 42.2 | 0 |
|  | **6. Chest tight** | 0.9 (0.8) | 0–4 | 32.6 | 0.3 | 0.7 (0.7) | 0–3 | 41.4 | 0 |
| **E-RS Breathlessness domain** | **7. Breathless** | 1.1 (0.8) | 0–3 | 21.7 | 0 | 1.0 (0.8) | 0–3 | 30.3 | 0 |
|  | **8. How breathless** | 1.1 (0.8) | 0–3 | 22.3 | 3.4 | 0.9 (0.8) | 0–3 | 30.3 | 2.3 |
|  | **9. Short of breath  with personal care** | 0.7 (0.8) | 0–4 | 48.5 | 0.3 | 0.5 (0.6) | 0–3 | 60.5 | 0 |
|  | **10. Short of breath  with indoor** | 1.0 (0.8) | 0–3 | 31.7 | 2.1 | 0.7 (0.8) | 0–3 | 44.8 | 1.5 |
|  | **11. Short of breath  with outdoor** | 1.2 (0.9) | 0–3 | 22.3 | 5.8 | 1.0 (0.9) | 0–3 | 32.2 | 3.8 |
| **E-RS Overall domain scores** | **E-RS-Cough and Sputum** | 3.0 (1.8) | 0–9 | 14.6 | 0 | 2.4 (1.8) | 0–8 | 25.3 | 0 |
|  | **E-RS-Chest Symptoms** | 2.7 (2.0) | 0–9 | 20.7 | 0 | 2.2 (1.9) | 0–8 | 28.0 | 0 |
|  | **E-RS-Breathlessness** | 5.0 (3.3) | 0–15 | 13.7 | 0 | 4.1 (3.2) | 0–15 | 23.0 | 0 |
| **E-RS Total score** | **E-RS-Total** | 10.7 (6.1) | 0–29 | 6.1 | 0 | 8.7 (6.0) | 0–31 | 12.3 | 0 |
| **Wheeze item†** | **Wheeze item** | 0.9 (0.8) | 0–4 | 32.3 | 0.6 | 0.7 (0.8) | 0–4 | 45.6 | 1.2 |

*Most items were scored 0–5; some items were scored 0–3; †the wheeze item was separate item to the E-RS instrument. Correlations >0.40 and >0.70 were defined as moderate and strong, respectively.
E-RS, Evaluating Respiratory Symptoms in chronic obstructive pulmonary disease; RS, respiratory symptom; SD, standard deviation.

# Table S3. Exploratory factor analysis at Day -1 for E-RS with and without the wheeze item in the overall population (N=328)

|  | **E-RS and Wheeze** | | | **E-RS** | | |
| --- | --- | --- | --- | --- | --- | --- |
| **Item** | **Factor 1** | **Factor 2** | **Factor 3** | **Factor 1** | **Factor 2** | **Factor 3** |
| 1. Chest congested | 0.18 | 0.42 | 0.32 | 0.18 | 0.33 | 0.41 |
| 2. Cough | 0.01 | 0.65 | 0.14 | 0.01 | 0.15 | 0.63 |
| 3. Mucus when coughing | -0.08 | 0.84 | -0.11 | -0.08 | -0.11 | 0.85 |
| 4. Difficulty with mucus | 0.25 | 0.54 | 0.01 | 0.25 | 0.02 | 0.53 |
| 5. Chest discomfort | 0.04 | 0.06 | 0.86 | 0.03 | 0.878 | 0.06 |
| 6. Chest tight | 0.08 | -0.07 | 0.88 | 0.09 | 0.87 | -0.07 |
| 7. Breathless | 0.70 | -0.02 | 0.15 | 0.68 | 0.17 | -0.02 |
| 8. How breathless | 0.65 | -0.08 | 0.18 | 0.63 | 0.19 | -0.08 |
| 9. Short of breath with personal care | 0.62 | 0.05 | 0.12 | 0.62 | 0.12 | 0.06 |
| 10. Short of breath with indoor | 0.84 | 0.08 | -0.08 | 0.85 | -0.08 | 0.08 |
| 11. Short of breath with outdoor | 0.87 | 0.01 | -0.04 | 0.86 | -0.03 | 0.01 |
| Wheeze item | 0.36 | 0.26 | 0.12 | - | - | - |

Data are expressed as promax rotation factor loadings.
E-RS, Evaluating Respiratory Symptoms in chronic obstructive pulmonary disease.

# Table S4. Test-retest reliability of E-RS Total and domain scores and wheeze at Day -7 to Day -6*

|  | **Scale** | **Difference in mean (SD) score between days** | **p-value** | **ICC (95% CI)** |
| --- | --- | --- | --- | --- |
| All | RS-Total | -0.02 | 0.9011 | 0.83 (0.79–0.86) |
|  | RS-Cough and Sputum | -0.02 | 0.7061 | 0.78 (0.73–0.82) |
|  | RS-Chest Symptoms | -0.07 | 0.3715 | 0.75 (0.70–0.79) |
|  | RS-Breathlessness | 0.07 | 0.5394 | 0.81 (0.77–0.84) |
|  | Wheeze | 0.02 | 0.5591 | 0.67 (0.61–0.73) |
| Morning PEF change <15% | RS-Total | -0.09 | 0.6979 | 0.81 (0.77–0.85) |
|  | RS-Cough and Sputum | -0.11 | 0.1673 | 0.75 (0.68–0.80) |
|  | RS-Chest Symptoms | -0.06 | 0.5256 | 0.76(0.70–0.81) |
|  | RS-Breathlessness | 0.07 | 0.5773 | 0.79 (0.74–0.83) |
|  | Wheeze | 0 | 0.9194 | 0.65 (0.57–0.72) |

*For Days -7 to Day -6, n=322 for all patients and n=229 for morning PEF change <15%; Cronbach alpha and ICC values >0.70 were considered acceptable.

CI, confidence interval; E-RS, Evaluating Respiratory Symptoms in chronic obstructive pulmonary disease; ICC, intra-class correlations; PEF, peak expiratory flow, RS, respiratory symptom; SD, standard deviation.

# Table S5. Descriptive statistics for E-RS domain and Total scores and wheeze item score for Day -1 (asthma: N=179, COPD: N=149) and Day 28 (asthma: N=137, COPD: N=124) by primary diagnosis of asthma and COPD

|  |  |  | **Day -1** | | | | **Day 28** | | | |
| --- | --- | --- | --- | --- | --- | --- | --- | --- | --- | --- |
|  | **Item*** |  | **Mean (SD)** | **Observed range** | **Floor (%)** | **Ceiling (%)** | **Mean (SD)** | **Observed range** | **Floor (%)** | **Ceiling (%)** |
| **E-RS Cough and Sputum domain** | **1. Chest congested** | Asthma | 0.85 (0.70) | 0–3 | 32.40 | 0 | 0.72 (0.66) | 0–2 | 40.15 | 0 |
|  |  | COPD | 1.01 (0.79) | 0–3 | 26.17 | 0 | 0.78 (0.75) | 0–3 | 39.52 | 0 |
|  | **2. Cough** | Asthma | 1.08 (0.79) | 0–3 | 21.79 | 0 | 0.85 (0.71) | 0–3 | 32.12 | 0 |
|  |  | COPD | 1.28 (0.84) | 0–4 | 14.09 | 2.01 | 1.09 (0.82) | 0–3 | 24.19 | 0 |
|  | **3. Mucus when coughing** | Asthma | 0.74 (0.48) | 0–2 | 27.93 | 0 | 0.61 (0.49) | 0–1 | 39.42 | 0 |
|  |  | COPD | 0.83 (0.45) | 0–2 | 20.13 | 0 | 0.72 (0.49) | 0–2 | 29.84 | 0 |
|  | **4. Difficulty with mucus** | Asthma | 0.96 (0.84) | 0–4 | 31.28 | 0.56 | 0.74 (0.86) | 0–4 | 46.72 | 0.73 |
|  |  | COPD | 1.10 (0.91) | 0–4 | 27.52 | 1.34 | 0.85 (0.77) | 0–3 | 35.48 | 0 |
| **E-RS Chest Symptoms domain** | **5. Chest discomfort** | Asthma | 0.87 (0.71) | 0–3 | 31.84 | 0 | 0.70 (0.70) | 0–2 | 43.80 | 0 |
|  |  | COPD | 0.97 (0.82) | 0–4 | 29.53 | 0.67 | 0.76 (0.71) | 0–2 | 40.32 | 0 |
|  | **6. Chest tight** | Asthma | 0.85 (0.74) | 0–3 | 34.64 | 0 | 0.72 (0.74) | 0–3 | 43.80 | 0 |
|  |  | COPD | 0.94 (0.81) | 0–4 | 30.20 | 0.67 | 0.77 (0.72) | 0–3 | 38.71 | 0 |
| **E-RS Breathlessness domain** | **7. Breathless** | Asthma | 1.05 (0.75) | 0–3 | 24.02 | 0 | 0.89 (0.80) | 0–3 | 34.31 | 0 |
|  |  | COPD | 1.21 (0.80) | 0–3 | 18.79 | 0 | 1.02 (0.78) | 0–3 | 25.81 | 0 |
|  | **8. How breathless** | Asthma | 0.97 (0.71) | 0–3 | 24.58 | 2.23 | 0.83 (0.72) | 0–3 | 33.58 | 2.19 |
|  |  | COPD | 1.19 (0.80) | 0–3 | 19.46 | 4.70 | 1.02 (0.78) | 0–3 | 26.61 | 2.42 |
|  | **9. Short of breath  with personal care** | Asthma | 0.63 (0.73) | 0–3 | 50.84 | 0 | 0.42 (0.59) | 0–2 | 62.77 | 0 |
|  |  | COPD | 0.77 (0.86) | 0–4 | 45.64 | 0.67 | 0.52 (0.69) | 0–3 | 58.06 | 0 |
|  | **10. Short of breath  with indoor** | Asthma | 0.91 (0.76) | 0–3 | 32.96 | 1.12 | 0.65 (0.71) | 0–3 | 48.18 | 0.73 |
|  |  | COPD | 1.01 (0.83) | 0–3 | 30.20 | 3.36 | 0.80 (0.80) | 0–3 | 41.13 | 2.42 |
|  | **11. Short of breath  with outdoor** | Asthma | 1.11 (0.82) | 0–3 | 24.58 | 3.91 | 0.96 (0.85) | 0–3 | 34.31 | 3.65 |
|  |  | COPD | 1.33 (0.88) | 0–3 | 19.46 | 8.05 | 1.06 (0.86) | 0–3 | 29.84 | 4.03 |
| **E-RS Overall domain scores** | **RS-Cough and Sputum** | Asthma | 2.78 (1.79) | 0–8 | 18.99 | 0 | 2.20 (1.73) | 0–6 | 28.47 | 0 |
|  |  | COPD | 3.20 (1.71) | 0–9 | 9.40 | 0 | 2.66 (1.83) | 0–8 | 21.77 | 0 |
|  | **RS-Chest Symptoms** | Asthma | 2.57 (1.88) | 0–8 | 22.91 | 0 | 2.14 (1.89) | 0–7 | 29.93 | 0 |
|  |  | COPD | 2.92 (2.14) | 0–9 | 18.12 | 0 | 2.31 (1.93) | 0–8 | 25.81 | 0 |
|  | **RS-Breathlessness** | Asthma | 4.66 (3.13) | 0–15 | 14.53 | 0 | 3.75 (3.07) | 0–11 | 24.09 | 0 |
|  |  | COPD | 5.50 (3.45) | 0–15 | 12.75 | 0 | 4.43 (3.30) | 0–15 | 21.77 | 0 |
| **E-RS Total score** | **RS-Total score** | Asthma | 10.01 (5.91) | 0–25 | 7.26 | 0 | 8.09 (5.87) | 0–23 | 13.87 | 0 |
|  |  | COPD | 11.62 (6.27) | 0–29 | 4.7 | 0 | 9.40 (6.13) | 0–31 | 10.48 | 0 |
|  | **Wheeze item†** | Asthma | 0.93 (0.80) | 0–3 | 31.84 | 0 | 0.72 (0.80) | 0–4 | 47.45 | 0.73 |
|  |  | COPD | 0.95 (0.87) | 0–4 | 32.89 | 1.34 | 0.73 (0.81) | 0–4 | 43.55 | 1.61 |

*Most items were scored 0–5; some items were scored 0–3; †the wheeze item was separate item to the E-RS instrument. Items were re-scored from the patient completed scores; correlations >0.40 and >0.70 were defined as moderate and strong, respectively.

COPD, chronic obstructive pulmonary disease; E-RS, Evaluating Respiratory Symptoms in COPD; RS, respiratory symptom; SD, standard deviation.

# Table S6. Inter-item correlations* for E-RS and wheeze items for Day -1 in patients with asthma (N=179)

| **Variable** | **Chest congested** | **Cough** | **Mucus when coughing** | **Difficulty with mucus** | **Chest discomfort** | **Chest tight** | **Breathless** | **How breathless** | **Short of breath with personal care** | **Short of breath  with indoor** | **Short of breath with outdoor** | **Wheeze** |
| --- | --- | --- | --- | --- | --- | --- | --- | --- | --- | --- | --- | --- |
| 1. Chest congested | 1.0 | 0.6 | 0.5 | 0.6 | 0.6 | 0.6 | 0.5 | 0.5 | 0.5 | 0.5 | 0.5 | 0.6 |
| 2. Cough | 0.6 | 1.0 | 0.6 | 0.6 | 0.4 | 0.4 | 0.4 | 0.4 | 0.3 | 0.3 | 0.4 | 0.5 |
| 3. Mucus when coughing | 0.5 | 0.6 | 1.0 | 0.6 | 0.4 | 0.3 | 0.3 | 0.3 | 0.3 | 0.4 | 0.3 | 0.4 |
| 4. Difficulty with mucus | 0.6 | 0.6 | 0.6 | 1.0 | 0.5 | 0.5 | 0.5 | 0.5 | 0.4 | 0.5 | 0.5 | 0.5 |
| 5. Chest discomfort | 0.6 | 0.4 | 0.4 | 0.5 | 1.0 | 0.8 | 0.6 | 0.6 | 0.5 | 0.5 | 0.5 | 0.5 |
| 6. Chest tight | 0.6 | 0.4 | 0.3 | 0.5 | 0.8 | 1.0 | 0.6 | 0.6 | 0.5 | 0.5 | 0.5 | 0.5 |
| 7.Breathless | 0.5 | 0.4 | 0.3 | 0.5 | 0.6 | 0.6 | 1.0 | 0.8 | 0.5 | 0.5 | 0.7 | 0.5 |
| 8. How breathless | 0.5 | 0.4 | 0.3 | 0.5 | 0.6 | 0.6 | 0.8 | 1.0 | 0.4 | 0.5 | 0.6 | 0.6 |
| 9. Short of breath  with personal care | 0.5 | 0.3 | 0.3 | 0.4 | 0.5 | 0.5 | 0.5 | 0.4 | 1.0 | 0.7 | 0.6 | 0.4 |
| 10. Short of breath  with indoor | 0.5 | 0.3 | 0.4 | 0.5 | 0.5 | 0.5 | 0.5 | 0.5 | 0.7 | 1.0 | 0.7 | 0.4 |
| 11. Short of breath  with outdoor | 0.5 | 0.4 | 0.3 | 0.5 | 0.5 | 0.5 | 0.7 | 0.6 | 0.6 | 0.7 | 1.0 | 0.5 |
| Wheeze | 0.6 | 0.5 | 0.4 | 0.5 | 0.5 | 0.5 | 0.5 | 0.6 | 0.4 | 0.4 | 0.5 | 1.0 |

*Data were analyzed using Spearman’s correlation; boxes depict correlations occurring within the same domain of the E-RS questionnaire; factor loadings >0.40 were considered acceptable.
E-RS, Evaluating Respiratory Symptoms in chronic obstructive pulmonary disease.

# Table S7. Inter-item correlations* for E-RS and wheeze items for Day -1 in patients with COPD (N=149)

| **Variable** | **Chest congested** | **Cough** | **Mucus when coughing** | **Difficulty with mucus** | **Chest discomfort** | **Chest tight** | **Breathless** | **How breathless** | **Short of breath with personal care** | **Short of breath  with indoor** | **Short of breath with outdoor** | **Wheeze** |
| --- | --- | --- | --- | --- | --- | --- | --- | --- | --- | --- | --- | --- |
| 1. Chest congested | 1.0 | 0.5 | 0.4 | 0.5 | 0.6 | 0.6 | 0.4 | 0.4 | 0.5 | 0.5 | 0.5 | 0.5 |
| 2. Cough | 0.5 | 1.0 | 0.4 | 0.5 | 0.5 | 0.3 | 0.4 | 0.3 | 0.4 | 0.5 | 0.4 | 0.4 |
| 3. Mucus when coughing | 0.4 | 0.4 | 1.0 | 0.4 | 0.2 | 0.1 | 0.1 | 0.0 | 0.2 | 0.2 | 0.2 | 0.2 |
| 4. Difficulty with mucus | 0.5 | 0.5 | 0.4 | 1.0 | 0.4 | 0.3 | 0.4 | 0.3 | 0.4 | 0.5 | 0.4 | 0.4 |
| 5. Chest discomfort | 0.6 | 0.5 | 0.2 | 0.4 | 1.0 | 0.8 | 0.5 | 0.4 | 0.5 | 0.5 | 0.5 | 0.5 |
| 6. Chest tight | 0.6 | 0.3 | 0.1 | 0.3 | 0.8 | 1.0 | 0.5 | 0.5 | 0.5 | 0.5 | 0.5 | 0.4 |
| 7.Breathless | 0.4 | 0.4 | 0.1 | 0.4 | 0.5 | 0.5 | 1.0 | 0.7 | 0.5 | 0.6 | 0.7 | 0.4 |
| 8. How breathless | 0.4 | 0.3 | 0.0 | 0.3 | 0.4 | 0.5 | 0.7 | 1.0 | 0.5 | 0.6 | 0.6 | 0.4 |
| 9. Short of breath  with personal care | 0.5 | 0.4 | 0.2 | 0.4 | 0.5 | 0.5 | 0.5 | 0.5 | 1.0 | 0.7 | 0.5 | 0.5 |
| 10. Short of breath  with indoor | 0.5 | 0.5 | 0.2 | 0.5 | 0.5 | 0.5 | 0.6 | 0.6 | 0.7 | 1.0 | 0.7 | 0.5 |
| 11. Short of breath  with outdoor | 0.5 | 0.4 | 0.2 | 0.4 | 0.5 | 0.5 | 0.7 | 0.6 | 0.5 | 0.7 | 1.0 | 0.4 |
| Wheeze | 0.5 | 0.4 | 0.2 | 0.4 | 0.5 | 0.4 | 0.4 | 0.4 | 0.5 | 0.5 | 0.4 | 1.0 |

*Data were analyzed using Spearman’s correlation; boxes depict correlations occurring within the same domain of the E-RS questionnaire; factor loadings >0.40 were considered acceptable.
COPD, chronic obstructive pulmonary disease; E-RS, Evaluating Respiratory Symptoms in COPD.

# Table S8. Confirmatory factor analysis of the E-RS at Day -1 by primary diagnosis of asthma (N=179)

| **Domain** | **Item** | **Factor loadings** | | | **Second order factor loading** |
| --- | --- | --- | --- | --- | --- |
|  |  | **Factor 1** | **Factor 2** | **Factor 3** |  |
| **Cough and Sputum** | 2. Cough | 0.77 (0.04) |  |  | 0.72 (0.06) |
|  | 3. Mucus when coughing | 0.73 (0.05) |  |  |  |
|  | 4. Difficulty with mucus | 0.77 (0.04) |  |  |  |
| **Chest Symptoms** | 1. Chest congested |  | 0.71 (0.04) |  | 0.91 (0.05) |
|  | 5. Chest discomfort |  | 0.90 (0.02) |  |  |
|  | 6. Chest tight |  | 0.84 (0.03) |  |  |
| **Breathlessness** | 7. Breathless |  |  | 0.81 (0.03) | 0.85 (0.05) |
|  | 8. How breathless |  |  | 0.74 (0.04) |  |
|  | 9. Short of breath with personal care |  |  | 0.73 (0.04) |  |
|  | 10. Short of breath with indoor |  |  | 0.79 (0.03) |  |
|  | 11. Short of breath with outdoor |  |  | 0.84 (0.03) |  |
| **Model fitting Statistics** | |  |  |  |  |
| Comparative fit index | |  |  |  | 0.90 |
| Standardized root mean square residual | |  |  |  | 0.07 |
| Root mean square error of approximation | |  |  |  | 0.13 |

Data are displayed as the correlation coefficient r (p-value). Factor loadings >0.40 were considered acceptable.
E-RS, Evaluating Respiratory Symptoms in chronic obstructive pulmonary disease.

# Table S9. Confirmatory factor analysis of the E-RS at Day -1 by primary diagnosis of COPD (N=149)

| **Domain** | **Item** | **Factor loadings** | | | **Second order factor loading** |
| --- | --- | --- | --- | --- | --- |
|  |  | **Factor 1** | **Factor 2** | **Factor 3** |  |
| **Cough and Sputum** | 2. Cough | 0.74 (0.06) |  |  | 0.77 (0.07) |
|  | 3. Mucus when coughing | 0.46 (0.08) |  |  |  |
|  | 4. Difficulty with mucus | 0.64 (0.07) |  |  |  |
| **Chest Symptoms** | 1. Chest congested |  | 0.66 (0.05) |  | 0.77 (0.06) |
|  | 5. Chest discomfort |  | 0.94 (0.02) |  |  |
|  | 6. Chest tight |  | 0.90 (0.02) |  |  |
| **Breathlessness** | 7. Breathless |  |  | 0.80 (0.04) | 0.89 (0.06) |
|  | 8. How breathless |  |  | 0.72 (0.04) |  |
|  | 9. Short of breath with personal care |  |  | 0.73 (0.04) |  |
|  | 10. Short of breath with indoor |  |  | 0.85 (0.03) |  |
|  | 11. Short of breath with outdoor |  |  | 0.81 (0.03) |  |
| **Model fitting Statistics** | |  |  |  |  |
| Comparative fit index | |  |  |  | 0.90 |
| Standardized root mean square residual | |  |  |  | 0.09 |
| Root mean square error of approximation | |  |  |  | 0.12 |

Data are displayed as the correlation coefficient r (p-value). Factor loadings >0.40 were considered acceptable.
COPD, chronic obstructive pulmonary disease; E-RS, Evaluating Respiratory Symptoms in COPD.

# Table S10. Exploratory factor analysis at Day -1 for E-RS, with and without the wheeze item, in patients with asthma (N=179)

|  | **E-RS and Wheeze** | | | **E-RS** | | |
| --- | --- | --- | --- | --- | --- | --- |
| **Item** | **Factor 1** | **Factor 2** | **Factor 3** | **Factor 1** | **Factor 2** | **Factor 3** |
| 1. Chest congested | 0.12 | 0.35 | 0.42 | 0.13 | 0.35 | 0.42 |
| 2. Cough | -0.10 | 0.07 | 0.81 | -0.09 | 0.074 | 0.80 |
| 3. Mucus when coughing | 0.01 | -0.10 | 0.82 | 0.02 | -0.10 | 0.82 |
| 4. Difficulty with mucus | 0.18 | 0.07 | 0.60 | 0.18 | 0.07 | 0.60 |
| 5. Chest discomfort | 0.02 | 0.83 | 0.06 | 0.02 | 0.84 | 0.06 |
| 6. Chest tight | -0.05 | 0.93 | -0.04 | -0.03 | 0.91 | -0.03 |
| 7. Breathless | 0.38 | 0.49 | -0.01 | 0.39 | 0.48 | -0.01 |
| 8. How breathless | 0.33 | 0.47 | 0.01 | 0.33 | 0.45 | 0.01 |
| 9. Short of breath with personal care | 0.75 | 0.03 | 0.01 | 0.75 | 0.03 | 0.021 |
| 10. Short of breath with indoor | 0.89 | -0.0458 | 0.02 | 0.89 | -0.05 | 0.03 |
| 11. Short of breath with outdoor | 0.77 | 0.13 | -0.01 | 0.76 | 0.13 | 0 |
| Wheeze item | 0.22 | 0.31 | 0.26 | - | - | - |

Data are expressed as promax rotation factor loadings.
E-RS, Evaluating Respiratory Symptoms in chronic obstructive pulmonary disease.

# Table S11. Exploratory factor analysis at Day -1 for E-RS, with and without the wheeze item, in patients with COPD (N=149)

|  | **E-RS and Wheeze** | | | **E-RS** | | |
| --- | --- | --- | --- | --- | --- | --- |
| **Item** | **Factor 1** | **Factor 2** | **Factor 3** | **Factor 1** | **Factor 2** | **Factor 3** |
| 1. Chest congested | 0.16 | 0.37 | 0.43 | 0.18 | 0.38 | 0.40 |
| 2. Cough | 0.11 | 0.20 | 0.51 | 0.13 | 0.212 | 0.49 |
| 3. Mucus when coughing | -0.15 | -0.11 | 0.81 | -0.14 | -0.10 | 0.84 |
| 4. Difficulty with mucus | 0.30 | -0.01 | 0.46 | 0.32 | 0.01 | 0.43 |
| 5. Chest discomfort | -0.02 | 0.92 | 0.06 | -0.03 | 0.94 | 0.05 |
| 6. Chest tight | 0.07 | 0.93 | -0.12 | 0.08 | 0.91 | -0.101 |
| 7. Breathless | 0.82 | 0.02 | -0.04 | 0.80 | 0.034 | -0.04 |
| 8. How breathless | 0.77 | 0.09 | -0.19 | 0.75 | 0.10 | -0.18 |
| 9. Short of breath with personal care | 0.49 | 0.26 | 0.10 | 0.50 | 0.27 | 0.08 |
| 10. Short of breath with indoor | 0.79 | -0.03 | 0.14 | 0.81 | -0.03 | 0.14 |
| 11. Short of breath with outdoor | 0.80 | -0.03 | 0.06 | 0.81 | -0.03 | 0.07 |
| Wheeze item | 0.35 | 0.10 | 0.30 | - | - | - |

Data are expressed as promax rotation factor loadings.
COPD, chronic obstructive pulmonary disease; E-RS, Evaluating Respiratory Symptoms in COPD.

# Table S12. The known groups validity of E-RS Total and domain scores and wheeze item at the baseline week in patients with asthma

| **Subgroup variable** | **Subgroup** | **n** | **RS-Total** | **RS-Breathlessness** | **RS-Cough and Sputum** | **RS-Chest Symptoms** | **Wheeze** |
| --- | --- | --- | --- | --- | --- | --- | --- |
| Exacerbation history  (number) | None, mean (SD) | 110 | 9.89 (5.32) | 4.56 (2.84) | 2.74 (1.48) | 2.59 (1.69) | 0.87 (0.70) |
|  | ≥1, mean (SD) | 73 | 10.04 (5.49) | 4.70 (2.97) | 2.73 (1.54) | 2.61 (1.61) | 0.94 (0.68) |
|  | F value |  | 0.0346 | 0.1083 | 0.0033 | 0.0066 | 0.4577 |
|  | p-value |  | 0.8527 | 0.7425 | 0.9545 | 0.9352 | 0.4996 |
| FEV_1_ % predicted  (Day -28) | <50%, mean (SD) | 70 | 10.98 (5.43) | 5.33 (2.95) | 2.84 (1.55) | 2.81 (1.70) | 1.02 (0.69) |
|  | 50–80%, mean (SD) | 113 | 9.31 (5.26) | 4.18 (2.77) | 2.67 (1.47) | 2.46 (1.62) | 0.82 (0.69) |
|  | F value |  | 4.2511 | 7.0675 | 0.5942 | 1.8929 | 3.5655 |
|  | p-value |  | 0.0407 | 0.0086 | 0.4418 | 0.1706 | 0.0606 |
| FEV_1_ % predicted  (Day 1) | <50%, mean (SD) | 42 | 11.53 (4.73) | 5.84 (2.74) | 2.91 (1.49) | 2.79 (1.59) | 1.10 (0.67) |
|  | 50–80%, mean (SD) | 139 | 9.48 (5.50) | 4.27 (2.85) | 2.68 (1.51) | 2.53 (1.67) | 0.84 (0.69) |
|  | ≥80%, mean (SD) | 2 | 8.89 (5.10) | 3.23 (1.33) | 2.85 (1.40) | 2.82 (2.37) | 0.75 (1.06) |
|  | F value |  | 2.4298 | 5.234 | 0.3827 | 0.3912 | 2.246 |
|  | p-value |  | 0.0909 | 0.0062 | 0.6826 | 0.6768 | 0.1088 |
| mMRC dyspnea status | 0–1, mean (SD) | 75 | 8.64 (5.09) | 3.98 (2.62) | 2.39 (1.48) | 2.27 (1.63) | 0.72 (0.59) |
|  | 2, mean (SD) | 99 | 10.73 (5.46) | 4.95 (2.88) | 2.97 (1.50) | 2.82 (1.68) | 1.02 (0.73) |
|  | 3–4, mean (SD) | 9 | 12.16 (4.76) | 6.22 (4.05) | 3.08 (1.26) | 2.86 (1.14) | 1.12 (0.75) |
|  | F value |  | 4.1744 | 3.995 | 3.5234 | 2.4476 | 4.5148 |
|  | p-value |  | 0.0169 | 0.0201 | 0.0316 | 0.0894 | 0.0122 |

The baseline week was defined as the last week of run-in (Day -7 to Day -1).
COPD, chronic obstructive pulmonary disease; E-RS, Evaluating Respiratory Symptoms in COPD; FEV_1_, forced expiratory volume in 1 second; mMRC, Modified Medical Research Council; RS, respiratory symptoms; SD, standard deviation.

# Table S13. The known groups validity of E-RS Total and domain scores and wheeze item at the baseline week in patients with COPD

| **Subgroup variable** | **Subgroup** | **n** | **RS-Total** | **RS-Breathlessness** | **RS-Cough and Sputum** | **RS-Chest Symptoms** | **Wheeze** |
| --- | --- | --- | --- | --- | --- | --- | --- |
| Exacerbation history  (number) | None, mean (SD) | 95 | 11.29 (6.36) | 5.39 (3.34) | 3.21 (1.78) | 2.69 (1.93) | 0.87 (0.83) |
|  | ≥1, mean (SD) | 60 | 12.49 (4.87) | 6.04 (2.71) | 3.31 (1.32) | 3.14 (1.56) | 1.10 (0.71) |
|  | F value |  | 1.5437 | 1.5729 | 0.1345 | 2.3403 | 3.219 |
|  | p-value |  | 0.216 | 0.2117 | 0.7144 | 0.1281 | 0.0748 |
| FEV_1_ % predicted  (Day -28) | <50%, mean (SD) | 72 | 12.10 (5.74) | 6.00 (3.01) | 3.15 (1.64) | 2.95 (1.81) | 0.96 (0.77) |
|  | 50–80%, mean (SD) | 83 | 11.45 (5.94) | 5.34 (3.20) | 3.33 (1.60) | 2.78 (1.80) | 0.97 (0.81) |
|  | F value |  | 0.4778 | 1.7205 | 0.4784 | 0.3567 | 0.0054 |
|  | p-value |  | 0.4905 | 0.1916 | 0.4902 | 0.5512 | 0.9415 |
| FEV_1_ % predicted  (Day 1) | <50%, mean (SD) | 60 | 12.52 (5.76) | 6.30 (3.04) | 3.38 (1.74) | 2.83 (1.76) | 1.01 (0.78) |
|  | 50–80%, mean (SD) | 95 | 11.27 (5.87) | 5.23 (3.11) | 3.17 (1.53) | 2.88 (1.83) | 0.93 (0.79) |
|  | F value |  | 1.6944 | 4.4993 | 0.672 | 0.0246 | 0.3958 |
|  | p-value |  | 0.195 | 0.0355 | 0.4136 | 0.8757 | 0.5302 |
| mMRC dyspnea status | 0–1, mean (SD) | 34 | 10.14 (5.91) | 4.83 (2.93) | 2.98 (1.57) | 2.33 (1.87) | 0.70 (0.73) |
|  | 2, mean (SD) | 98 | 11.51 (5.86) | 5.45 (3.18) | 3.16(1.64) | 2.91 (1.79) | 0.97 (0.76) |
|  | 3–4, mean (SD) | 23 | 15.17 (4.30) | 7.68 (2.25) | 4.03 (1.38) | 3.45 (1.58) | 1.31 (0.86) |
|  | F value |  | 5.6252 | 6.7298 | 3.4391 | 2.8183 | 4.3369 |
|  | p-value |  | 0.0044 | 0.0016 | 0.0346 | 0.0628 | 0.0147 |

The baseline week was defined as the last week of run-in (Day -7 to Day -1).
COPD, chronic obstructive pulmonary disease; E-RS, Evaluating Respiratory Symptoms in COPD; FEV_1_, forced expiratory volume in 1 second; mMRC, Modified Medical Research Council; RS, respiratory symptoms; SD, standard deviation.

# References

1. Celli BR, MacNee W, Force AET. Standards for the diagnosis and treatment of patients with COPD: a summary of the ATS/ERS position paper. Eur Respir J 2004;23:932-946.

2. Lee L, Kerwin E, Collison K, Nelsen L, Wu W, Yang S, Pascoe S. The effect of umeclidinium on lung function and symptoms in patients with fixed airflow obstruction and reversibility to salbutamol: A randomised, 3-phase study. Respir Med 2017;131:148-157.
